# Liver-specific deletion of *Ppp2cα* enhances glucose metabolism and insulin sensitivity

**DOI:** 10.18632/aging.100725

**Published:** 2015-02-21

**Authors:** Li Xian, Siyuan Hou, Zan Huang, An Tang, Peiliang Shi, Qinghua Wang, Anying Song, Shujun Jiang, Zhaoyu Lin, Shiying Guo, Xiang Gao

**Affiliations:** ^1^ Key Laboratory of Model Animal for Disease Study of Ministry of Education, Model Animal Research Center, Nanjing University, Nanjing, China

**Keywords:** PP2A, glucose, insulin signaling, liver, disorder

## Abstract

Protein phosphatase 2A (PP2A) is a key negative regulator of phosphatidylinositol 3-kinase/Akt pathway. Previous study showed that, in the liver, the catalytic subunit of PP2A (PP2Ac) is closely associated with insulin resistance syndrome, which is characterized by glucose intolerance and dyslipidemia. Here we studied the role of liver PP2Ac in glucose metabolism and evaluated whether PP2Ac is a suitable therapeutic target for treating insulin resistance syndrome. Liver-specific *Ppp2cα* knockout mice (*Ppp2cα*^loxp/loxp^: Alb) exhibited improved glucose homeostasis compared with littermate controls in both normal and high-fat diet conditions, despite no significant changes in body weight and liver weight under chow diet. *Ppp2cα*^loxp/loxp^: Alb mice showed enhanced glycogen deposition, serum triglyceride, cholesterol, low density lipoprotein and high density lipoprotein, activated insulin signaling, decreased expressions of gluconeogenic genes *G_6_P* and *PEPCK*, and lower liver triglyceride. Liver-specific *Ppp2cα* knockout mice showed enhanced glucose homeostasis and increased insulin sensitivity by activation of insulin signaling through Akt. These findings suggest that inhibition of hepatic *Ppp2cα* may be a useful strategy for the treatment of insulin resistance syndrome.

## INTRODUCTION

Low insulin sensitivity is referred to as insulin resistance, in which insulin fails to efficiently modulate glucose uptake, production, and storage in insulin-sensitive tissues [1, 2]. Hepatic insulin resistance is a key factor in the pathogenesis of insulin resistance syndrome, which is characterized by obesity, type 2 diabetes, coronary artery disease, and so on [3]. In recent years, researchers have got a clear picture of insulin-signaling network based on various considerable data, beginning with the binding of insulin-receptor tyrosine kinase and phosphorylation of the insulin-receptor substrates (IRS1 and IRS2). The activated receptor initiates a linear signaling cascade by phosphorylation of downstream target proteins [4-8]. Among the several components of the insulin-signaling network, phosphorylation of phosphatidylinositol 3-kinase (PI3K)/Akt is a critical node that regulates most actions of insulin [9-11]. Impairment of insulin signaling can lead to insulin resistance syndrome [12, 13].

Protein phosphatase 2A (PP2A) is one of the most abundant serine/threonine phosphatases that plays an important role in the regulation of many proteins, including metabolic enzymes, hormone receptors, kinase cascades, and cell growth factor [14-17]. Several studies reveal that PP2A is involved in the metabolic actions of insulin. Okadaic acid, an inhibitor of PP2A, can activate glucose transport and GLUT4 translocation [18]. Expression of small t antigen has been demonstrated that inhibiting PP2A in 3T3-L1 adipocytes also stimulates GLUT4 translocation and glucose transport [19]. Inhibition of FFAs in hepato-cytes from ZDF rats can cause hepatic insulin resistance by increasing PP2A activity, which reduces Akt-mediated gene expression [20]. Hyperglycemia and insulin resistance are also induced by chronic hepatitis C virus infection through overexpression of PP2A thereby inhibiting Akt signaling [21, 22]. These conclusions raised the possibility that PP2A is a key regulator of insulin signaling in the liver and targeting PP2A may have therapeutic benefit in treating insulin resistance.

PP2Ac is known as the catalytic subunit and is encoded by two distinct genes, *Ppp2cα* and *Ppp2cβ*. In all cases, *Ppp2cα* is 10-fold more abundant than *Ppp2cβ* [23], presumably due to different promoter activities. Unfortunately, complete loss of *Ppp2cα* results in early embryonic lethality at stage E6.5 in mice [24] and the mice lacking *Ppp2cβ* seem to be normal [25], which prevents analysis of the direct function of *Ppp2cα* in insulin resistance. In this study, we have generated conditional knockout *Ppp2cα* by disrupting the *Ppp2cα* gene in mouse liver to explore the effects of PP2A on hepatic control of glucose homeostasis and lipid metabolism.

## RESULTS

### Characterization of liver-specific *Ppp2cα* mice

**Figure 1:**
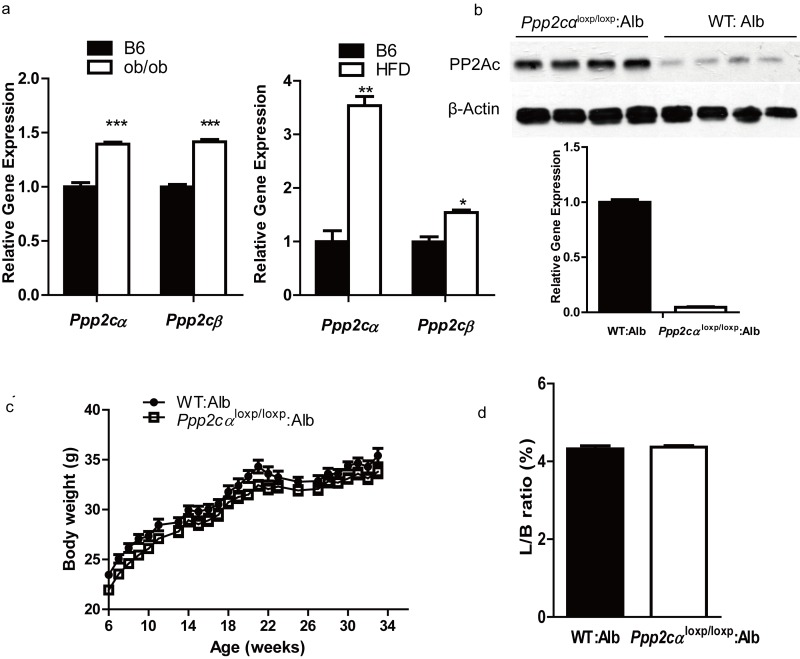
*Ppp2cα* is highly expressed in two models of insulin resistant and liver-specific deletion of *Ppp2cα*. **(a)** Q-PCR was performed to measure *Ppp2cα* mRNA levels in liver from ob/ob of 8weeks and 16 weeks mice fed HFD beginning at 8 weeks of age compared to appropriate controls (n=5). **(b)** Deletion efficiency of the *Ppp2cα* allele was analyzed using Q-PCR and western blotting. **(c)** Body weight (BW) of mice on a normal chow diet (n=8). BW was monitored every week from 6 weeks of age for 34 weeks. **(d)** Liver weight (normalized to BW) of 8-week-old mice fed ad libitum (n=8).

Previous work had indicated that PP2A has a negative effect on insulin metabolic signaling. To investigate more details about the association between *Ppp2cα* and insulin resistance in the liver, we first examined the expression of *Ppp2cα* in the livers of two mice models of obesity and type 2 diabetes, leptin-deficient ob/ob and high-fat diet (HFD) mice. As shown in Figure 1a, *Ppp2cα* mRNA level was significantly increased in these two models. Because total body loss of PP2Ac in mice results in early embryonic lethality at E6.5 phenotype, we generated mice with liver-specific *Ppp2cα* deletion by crossing *Ppp2cα*^fl/fl^ mice with Alb:Cre mice, which specifically expressed Cre recombinase in the liver. Because *Ppp2cα* and *Ppp2cβ* share a 97% identity in their amino acid sequence, the PP2Ac antibody cannot distinguish these two isoforms of the C subunit. Therefore, we used RT-PCR to confirm the deletion of *Ppp2cα* allele in the liver (Figure 1b).

Liver-specific *Ppp2cα* knockout (*Ppp2cα*^loxp/loxp^: Alb) mice were viable, born at the expected Mendelian frequency, and showed similar morphology to that of the control (wt: Alb) littermates. Next we examined the metabolic parameters of *Ppp2cα*^loxp/loxp^: Alb and control mice fed with normal chow and HFD. Loss of hepatic *Ppp2cα* displayed no difference of body weight in mice fed on a normal chow diet (Figure 1c). Moreover, loss of *Ppp2cα* did not affect the ratio of liver weight and body weight. Hematoxylin and eosin staining of liver sections revealed normal histology in both *Ppp2cα*^loxp/loxp^: Alb and control littermates (data not shown). Interestingly, lipoprotein analysis showed that *Ppp2cα*^loxp/loxp^: Alb mice fed on both chow diet and HFD had substantially higher serum TG, CHOL, LDL, and HDL (Table1).

**Table 1:** Quantification of metabolic parameters in *Ppp2cα*^loxp/loxp^: Alb and WT: Alb mice

	Normal chow	HFD
	WT: Alb Ppp2cα^loxp/loxp^: Alb	WT: Alb Ppp2cα^loxp/loxp^: Alb
Chol (mmol/l)	3.08±0.1 4.28±0.23**	3.56±0.56 5.6±0.22*
TG (mmol/l)	0.91±0.14 1.14±0.08	0.29±0.04 0.5±0.13
HDL-C (mmol/l)	2.18±0.07 2.94±0.14***	2.7±0.2 3.45±0.24*
LDL-C (mmol/l)	0.33±0.02 0.61±0.06**	0.8±0.1 1.26±0.13*

Averages were calculated (n=7-8 per group) and include standard deviations. Data were analyzed by two-tailed Student's *t* test. * indicates statistical significance from control mice

* p < 0.05

** p < 0.01.

### Improved glucose homeostasis in *Ppp2cα*^loxp/loxp^: Alb mice

**Figure 2:**
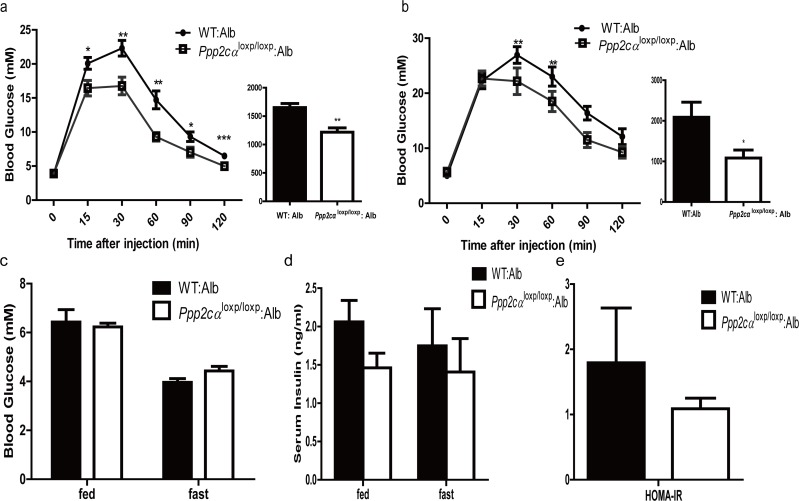
Deletion of *Ppp2cα* in the liver improves glucose tolerance (**a,b**) GTT on male *Ppp2cα*^loxp/loxp^: Alb and control mice (n=7-8) on chow diet (**a**) at 8 weeks of age and on HFD (**b**) for 8 weeks (16 weeks of age). Bar graphs to the right show the respective area under the curve (AUC) of glucose. (**c**) Fed and fasting serum glucose levels. (**d**) Fed and fasting serum insulin levels. (**e**) ALT activity. *Ppp2cα*^loxp/loxp^: Alb and WT: Alb control groups are indicated in the figures. Data are represented as mean ± SEM. Data were analyzed using two-tailed Student's t test (*p < 0.05, **p < 0.01, ***p < 0.001).

To assess whether *Ppp2cα* loss in the liver can affect whole body glucose homeostasis, we next performed GTT and ITT. Prior to injection of glucose and insulin, we measured blood glucose level and insulin levels in fasted and random fed mice groups. Despite the comparable body weight, liver weight: body weight ratios, and histology, *Ppp2cα*^loxp/loxp^: Alb mice showed enhanced ability to clear intraperitoneal glucose load from the peripheral circulation compared with littermate controls fed on normal chow and HFD (Figure 2a, b), as evidenced by a decreased AUC in *Ppp2cα*^loxp/loxp^: Alb mice. In comparison, the mice with loss of *Ppp2cα*^loxp/loxp^ showed comparable response after inulin perfusion on normal chow and HFD (data not shown). We then investigated basal blood glucose level under fasting and random fed conditions. However, there were no differences in plasma glucose levels between *Ppp2cα*^loxp/loxp^: Alb mice and control mice (Figure 2c). In addition, basal serum insulin levels were slightly decreased in *Ppp2cα*^loxp/loxp^: Alb mice (Figure 2d). No significant changes were detected in serum ALT levels between the groups (Figure 2e). These data indicated that inactivation of *Ppp2cα* in the liver leads to improved glucose tolerance, which may be due to enhanced insulin sensitivity in the liver.

### Increased glycogen deposition and decreased lipid homeostasis in *Ppp2cα*^loxp/loxp^: Alb mice

**Figure 3:**
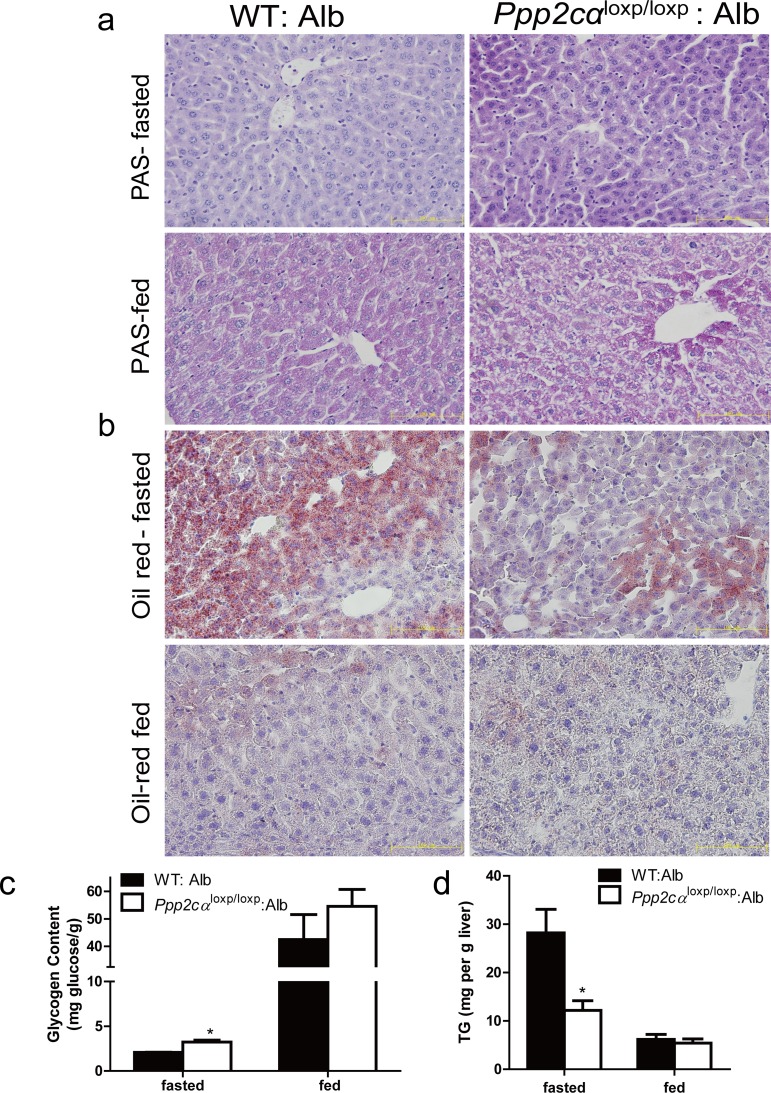
Loss of *Ppp2cα* significantly alters metabolism. (**a**) Accumulation of glycogen was detected by PAS staining and (**b**) decrease of lipid droplets was detected by Oil Red staining (**c**) Hepatic glycogen content measured in liver and (**d**) Hepatic TG content of the liver of 10-week-old *Ppp2cα*^loxp/loxp^: Alb and littermate controls following fasting overnight or random fed. Data were analyzed using two-tailed Student's t test (*p < 0.05)

Impaired storage and utilization of glucose is an index of the pathophysiology of insulin resistance [28, 29]. Periodic acid Schiff (PAS) staining was used to analyze paraffin-embedded liver sections for glycogen storage from *Ppp2cα*^loxp/loxp^: Alb and control mice. The liver sections from the fasted ontrol mice displayed minimal glycogen content, whereas those from fasted *Ppp2cα*^loxp/loxp^: Alb mice exhibited elevated glycogen. The same trends of increased glycogen were observed in random fed mice (Figure 3a). In accordance with PAS staining, quantitative analysis of glycogen also showed that liver glycogen was enhanced in both fasted and random fed *Ppp2cα*^loxp/loxp^: Alb mice (Figure 3b). By contrast, histological analysis of liver sections from *Ppp2cα*^loxp/loxp^: Alb mice for lipid deposition using Oil Red O staining revealed depletion of lipid compared with WT: Alb mice. Therefore, we also quantified plasma triglyceride levels, which were significantly decreased in *Ppp2cα*^loxp/loxp^: Alb mice compared with control WT: Alb mice (Figure 3c).

### Increased insulin signaling in liver lacking *Ppp2cα*

**Figure 4:**
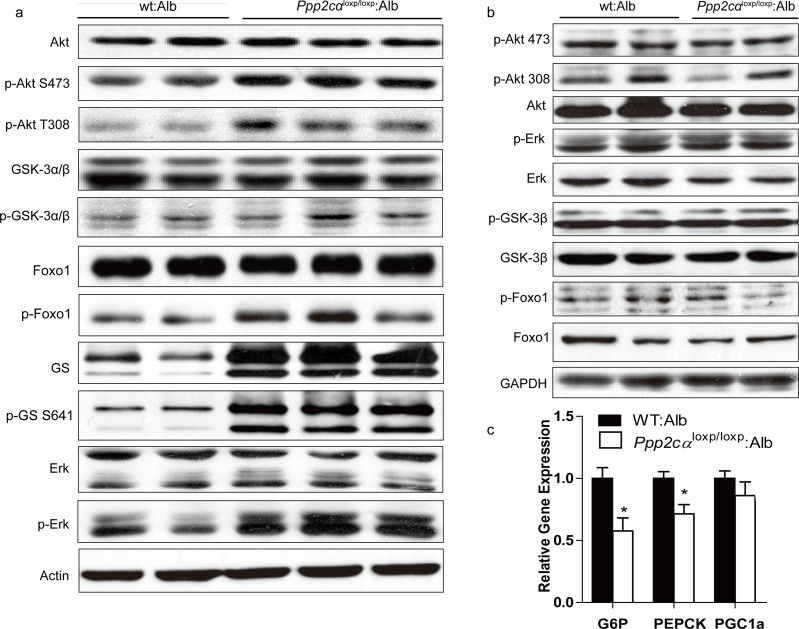
Enhanced insulin sensitivity in *Ppp2cα*^loxp/loxp^: Alb mice. (**a, b**) Western blot of insulin signaling involved Akt pathway in the liver (**a**) and muscle (**b**). Livers and skeletal muscle of overnight fasted mice on chow were isolated 5mins after 1U/kg insulin treatment. (**c**) Relative expression of *G_6_P*, *PEPCK*, *and PGC1α* mRNAs normalized against 36B4 mRNA levels, measured by Q-PCR in livers from fasted overnight mice. Data were analyzed by two-tailed Student's *t* test (*p < 0.05, ***p < 0.001).

PI3K/Akt is important for molecular and genetic studies on insulin signaling. To investigate the molecular mechanism contributing to improved glucose homeostasis, we examined insulin signaling by intra-peritoneal injection of insulin to fasted *Ppp2cα*^loxp/loxp^: Alb and WT: Alb mice. Besides, we also found that phosphorylation of Akt (Ser473, Thr308) was markedly higher in *Ppp2cα*^loxp/loxp^: Alb mice fed on chow diet compared with control WT: Alb mice, which was consistent with previous reports that PP2A can dephosphorylate and inactivate Akt. In addition, we showed that insulin-stimulated phosphorylation of Akt substrates GSK3α/β (Ser21/9) and Foxo1 (Ser264) was increased in the liver extracts from *Ppp2cα*^loxp/loxp^: Alb mice resulting in inactivation of GSK3α/β and Foxo1. As opposed to Akt activation, insulin treatment of the *Ppp2cα*^loxp/loxp^: Alb mice resulted in an increase of GS phosphorylation (Ser641). But GS was found to be more phosphorylated, and hence more inactive. At the moment, we also found total protein of GS was elevated (Figure 4a). Skeletal muscle is also recognized as a major tissue where insulin stimulates glucose use. Thus, insulin signaling in the muscle is critical for the regulation of whole body glucose homeostasis. Immunoblot analysis of skeletal muscle revealed no differences in the levels of Akt-insulin signaling between WT: Alb and *Ppp2cα*^loxp/loxp^: Alb mice (Figure 4b). Foxo1 is an important gluconeogenic transcription factor that affects mRNA levels of *G_6_P* and *PEPCK*. *G_6_P* and *PEPCK* mRNA levels were significantly decreased resulting in suppression of gluconeogenesis in the liver, which was consistent with activation of insulin signaling (Figure 4c).

### Wortmannin reverses the metabolic phenotypes

**Figure 5:**
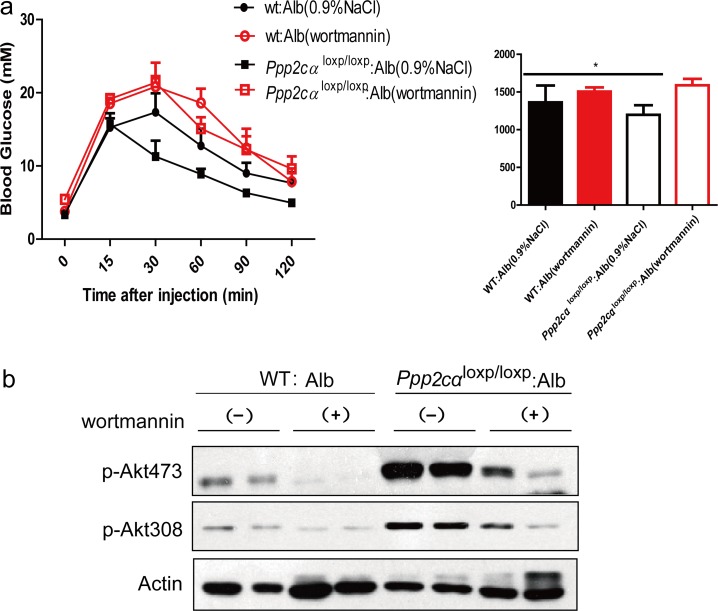
Effects of wortmannin on glucose of *Ppp2cα*^loxp/loxp^: Alb and WT: Alb mice. (**a**) GTT and AUC of over fasted *Ppp2cα*^loxp/loxp^: Alb and WT: Alb mice treated with wortmannin lasted 4weeks. (**b**) Immunoblot analysis with antibodies to Akt, phosphor-Akt (pSer^473^ and pThr^308^) was examined in *Ppp2cα*^loxp/loxp^: Alb and WT: Alb mice after injection with saline or insulin after 4 weeks wortmannin treatment. Data were analysed by two-tailed Student's *t* test (*p < 0.05).

According to the physiological and signaling data, if Akt is the primary target in PP2A-mediated metabolic regulation, inhibition of Akt should largely reverse the metabolic phenotype in these mice. Wortmannin is a common PI3K inhibitor that suppresses protein kinase B/Akt phosphorylation. To assess the effect of inhibition of Akt in the liver, we injected *Ppp2cα*^loxp/loxp^: Alb and WT: Alb mice with wortmannin. The efficient inhibition of Akt was confirmed by western blotting (Figure 5b). Moreover, we found that wortmannin treatment of *Ppp2cα*^loxp/loxp^: Alb mice prevented the improved glucose tolerance in control mice (Figure 5a). Taken together, these data support the conclusion that PP2Ac subunit regulates glucose homeostasis and its related metabolic events largely through Akt in the liver.

## DISCUSSION

Insulin resistance in the liver has been viewed as a central feature of the pathophysiology of metabolic disorders, including glucose intolerance, dyslipidemia, and insulin action. Recently, studies have demonstrated that PP2Ac is overexpressed in insulin resistance patients infected with chronic hepatitis C virus [21]. DNA microarray analysis of insulin-resistant rat induced by HFD showed that the expression of PP2A is upregulated [30]. Here we demonstrated that hepatic PP2Ac was highly expressed in two mice models of insulin resistance. In our study, we found mice with loss of *Ppp2cα* selectively in the liver and fed on chow diet and HFD had significantly improved ability to clear glucose from the peripheral circulation during GTT, whereas there were no changes in ITT between the two groups. Another physiological phenotype of *Ppp2cα* liver-specific deletion mice was enhancement of hepatic glycogen deposition in both fasted and fed mice.

As reported in other *in vitro* experimental systems, PP2A is a negative regulator of insulin metabolic signaling pathway, by dephosphorylation and inactivation of Akt [31]. Our study showed that *Ppp2cα*^loxp/loxp^: Alb mice displayed increased insulin-stimulated phosphorylation levels of Akt, GSK3α/β, Foxo1, and Erk. Whereas in the muscle, phosphorylation levels of these proteins were comparable between *Ppp2cα*^loxp/loxp^: Alb and WT: Alb mice. A well-defined pathway has been established– activated Akt phosphorylated Foxo1 after insulin stimulation, and inactivated Foxo1 suppressed the transcription of genes encoding gluconeogenic enzymes [9, 32-34]. Consistent with previous models, transcription levels of Foxo1-dependent genes, such as *G_6_P* and *Pepck*, were decreased. Reverse effects by wortamnnin inhibiting the phosphorylation of Akt on glucose homeostasis and insulin signaling suggested that PP2Ac directly influenced insulin signaling through Akt. In the liver, Akt is necessary for maintaining glucose homeostasis and insulin responsiveness. Indeed, hepatic ablation of Akt1 and Akt2 contributes to glucose tolerance and insulin resistance [10]. Akt2 deficiency in mice causes a mildly diabetes mellitus phenotype [6]. Further experiments using Akt-knockout mice crossed with *Ppp2cα-*knockout mice would be helpful to confirm this conclusion.

Previous reports support our results that PP2A participates in the response to inulin through Akt. However, as opposed to another previous study for hepatic PP2Ac function, rats treated with a small molecular inhibitor of PP2A (LB1) displayed hepatic insulin resistance and reduction of glycogen content [35]. The mechanism that accounts for the differential effects is unclear. The inhibitor was administered by intraperitoneal injection into the rats. It did not exclude the possibility that PP2A was inhibited in other tissues besides the liver. Moreover, 35% activity was inhibited after treatment by LB1. These factors of different targeting strategies may contribute to the differential phenotypes caused by deletion or inhibition of PP2A in the liver.

**Figure 6:**
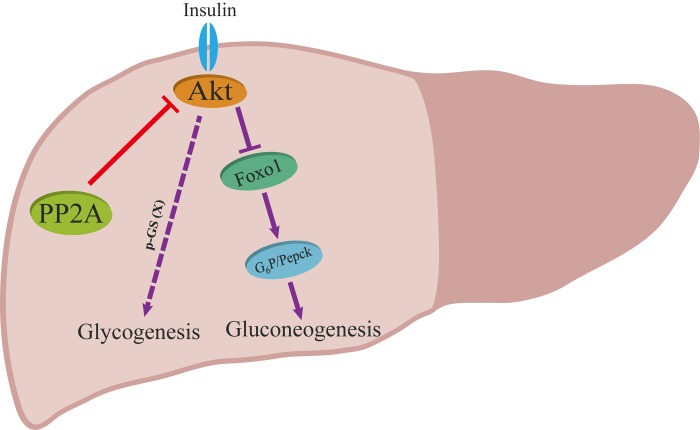
Proposed model for the role of PP2A on insulin signaling. Red and purple arrows illustrate the direct and indirect actions.

A generally accepted model includes the following observations: (i) insulin induces the activation and phosphorylation of Akt; (ii) activated Akt phosphorylates and suppresses GSK3; (iii) phosphorylated GSK3 inhibits the activity of GS through phosphorylation at Ser641 and finally promotes storage of glucose in the form of glycogen [36-38]. Interestingly, a phenotype was opposite to this canonical model in hepatic *Ppp2cα*–knockout mice, which displayed an increased level of phosphorylated GS, favoring storage of glucose in the liver, and hence it was thought that if GS was less phosphorylated, it would be more active. In the past several years, there have been some different views on the current knowledge about the canonical model. Previous work indicated that inactivation of GSK3 plays a minor role in glycogen storage in skeletal muscle, and G_6_P allostically regulating the activity of GS in response to insulin plays an important role in controlling glycogen synthesis in the skeletal muscle [39, 40]. Recent finding by Wan et al. demonstrated that GS activity did not correlate with phosphorylation of GS (Ser641), which is a direct target of GSK3 [41]. Here, we present genetic evidence supporting the idea that insulin signaling through Akt modulates the phosphorylation of GS at another possible residue after translation to control glycogen levels (Figure 6).

*Ppp2cα*^loxp/loxp^: Alb mice displayed lower liver triglyceride levels compared with control WT: Alb mice. This suggests that inhibition of PP2Ac may be a potential therapy for NAFLD, which is associated with pathologies, such as insulin resistance and increased hepatic lipid accumulation [42]. Nevertheless, we also found that serum CHOL, HDL, and LDL levels were significantly increased in *Ppp2cα*^loxp/loxp^: Alb mice. At the moment, it is unclear how hepatic PP2Ac knockout affects lipid and sterol metabolism.

In summary, liver-specific knockout studies of *Ppp2cα* show its key roles in maintaining metabolism of glucose, lipid, and cholesterol. We now demonstrate that hepatic *Ppp2cα* deletion not only enhances glucose homeostasis but can also increase insulin sensitivity by activation of Akt signaling. These findings provide new insight for inhibition of *Ppp2cα* as a therapeutic target for ameliorating insulin resistance syndrome.

## METHODS

### Animals

*Ppp2cα*^loxp/loxp^ mice were generated through homologous recombination as previously described [26]. Alb-Cre mice, when crossed to *Ppp2cα*^loxp/loxp^ mice, provide a tool to generate mice with liver specific deletion. Mice in this study were on a C57BL/6 genetic background and housed in groups with 12h dark/light cycles and free access to food and water in accordance with the regulations on mouse welfare and ethics of Nanjing University. All procedures were conducted with relevant authority approval. All experiments were conducted on male mice between 8 to 16 weeks of age on normal chow, and 16 to 20 weeks on HFD.

### Glucose and insulin tolerance tests

For glucose tolerance tests (GTT), mice were fasted overnight (16hr) then administrated with 2g/kg of body weight of glucose by intrperitoneal (i.p.) injection. For insulin tolerance tests (ITT), mice were fasted for 6hr before i.p. administration with 0.5IU/kg of body weight of insulin. Blood glucose concentrations were measured at the indicated time points.

### Histology

Liver from mice either fasted overnight or random fed were extracted and fixed in 4% paraforma-dehyde (PFA)/phosphate-buffered saline (PBS) overnight at 4°C. Paraffin sections were stained by PAS. Isolated liver was put in 4% PFA, equilibrated in 30% sucrose for 12h and then embedded in optimal cutting temperature compound (OCT). Frozen sections were stained with Oil Red O and counterstained with Hematoxylin.

### Immunoblotting

For assessment of insulin signaling in liver, animals were fasted overnight for 12h. Fasted mice were given an intrperitioneal injection of inulin (Novoli, 1U/kg body weight) and livers and muscles were removed 5 min later. Mouse tissues were frozen in liquid N2 immediately. Tissue lysates were prepared by extraction in radio immunoprecipitation assay (RIPA) buffer at 4°C, followed by clarification at 10,000 g. For immunoblots, samples were separated by SDS-PAGE and transferred to PVDF. Antibodies against phosphorylated Akt (Ser473 and Thr308), Akt, phosphorylated GSK3α/β (Ser21 and Ser9), Erk, phosphorylated Erk (Thr202/Tyr204), Foxo1, phosphorylated Foxo1 (Ser264), glycogen synthase, phosphorylated glycogen synthesis (Ser641), were purchased from Cell signaling Technology. β-actin antibody was purchased from Vazyme Biotech (Nanjing, China).

### Quantitative RT-PCR

Total RNA was extracted from fasted mouse liver using RNAIso Plus (Takara) and purified as manufacturer's protocol. A total of 1ug RNA was reversely transcribed into cDNA in 10ul reaction volume using the PrimeScript™ RT reagent Kit with gDNA Eraser (Takara). Then quantitative RT-PCR analysis were used to amplify the target genes with SYBR^®^ Premix Ex Taq™ (Takara) followed by reactions performed with an Applied Biosystems StepOne™ instrument. Relative gene expressions were calculated using the comparative Ct (2^−ßßCT^) methods normalized to housekeeping gene 36b4.

### Metabolic measurements

Blood glucose level in tail blood was measured with Glucacord II. Serum samples were collected from the retro-orbital plexus of mice. Centrifuge the clotted blood at 3,000g for 15 minutes at 4°C. Serum insulin was assayed by ELISA as manufacturers' instructions (Millipore). Concentrations of total triglycerides (TG), total cholesterol (CHOL), high-density lipoprotein (HDL), low-density lipoprotein (LDL) and alanine aminotrasnsferase (ALT) activity were determined by using an automated biochemical analyzer (Hitachi 7020) according to the manufacturers' instructions.

### Liver triglyceride

Isolated and weighted ~50mg liver were frozen in liquid N_2_ immediately. Liver triglycerides were extracted using the method of Jouihan [27]. Triacylglycerols were then assayed using a kit, following manufacturer's instructions (code no.290-63701; Wako).

### Wortmannin treatment in vivo

To prepare wortmannin, it was purchased from stored at −20°C in a 20mg/ml solution in DMSO and diluted with 0.9% NaCl before use. One group of mice was given via i.p. with wortmannin (2mg/kg) three times for 4weeks. The control mice were received with vehicle (0.9% NaCl). Wortmannin (s2758) was purchased from Selleck Chemicals.
